# Clinical Evaluation of Subcutaneous Lactate Measurement in Patients after Major Cardiac Surgery

**DOI:** 10.1155/2009/390975

**Published:** 2009-05-26

**Authors:** Martin Ellmerer, Martin Haluzik, Jan Blaha, Jaromir Kremen, Stepan Svacina, Andreas Plasnik, Dimas Ikeoka, Manfred Bodenlenz, Lukas Schaupp, Johannes Plank, Thomas R. Pieber

**Affiliations:** ^1^Department of Internal Medicine, Medical University Graz, Auenbruggerplatz 15, 8036 Graz, Austria; ^2^3rd Department of Medicine, 1st Faculty of Medicine, Charles University, Ruská 87, 100 00 Prague, Czech Republic; ^3^Joanneum Research GmbH, Institute of Medical System Technologies and Health Management, Elisabethstra*β*e 11a, 8010 Graz, Austria

## Abstract

Minimally invasive techniques to access subcutaneous adipose tissue for glucose monitoring are successfully applied in type1 diabetic and critically ill patients. During critical illness, the addition of a lactate sensor might enhance prognosis and early intervention. Our objective was to evaluate SAT as a site for lactate measurement in critically ill patients. In 40 patients after major cardiac surgery, arterial blood and SAT microdialysis samples were taken in hourly intervals. Lactate concentrations from SAT were prospectively calibrated to arterial blood. Analysis was based on comparison of absolute lactate concentrations (arterial blood vs. SAT) and on a 6-hour lactate trend analysis, to test whether changes of arterial lactate can be described by SAT lactate. Correlation between lactate readings from arterial blood vs. SAT was highly significant (*r*2 = 0.71, *P* < .001). Nevertheless, 42% of SAT lactate readings and 35% of the SAT lactate trends were not comparable to arterial blood. When a 6-hour stabilization period after catheter insertion was introduced, 5.5% of SAT readings and 41.6% of the SAT lactate trends remained incomparable to arterial blood. In conclusion, replacement of arterial blood lactate measurements by readings from SAT is associated with a substantial shortcoming in clinical predictability in patients after major cardiac surgery.

## 1. Introduction

The importance of lactate to monitor the metabolic stress response has been recognized long time ago in patients with circulatory failure [1]. In general, blood lactate has been considered as a marker of tissue hypoxia [2], while recent investigations also suggest that elevated levels of catecholamines play an important role in lactic acid production during acute diseases [3]. In the meanwhile, a large body of evidence from experimental and clinical studies established a tight association between hyperlactatemia and mortality in many diagnostic groups [4–9] and in 2001, Smith and colleagues were among the first using lactate levels as admission criteria for early intensive care medicine [8]. Nowadays, lactate levels are measured in critically ill patients on a routine basis using blood gas analysis and hyperlactatemia prompts clinicians to initiate further diagnostic and therapeutic actions. Both absolute levels and profiles (trend information) of the blood lactate concentration provide important information about the ongoing pathophysiological processes of critically ill patients [10].

Subcutaneous adipose tissue (SAT) has been suggested as a possible alternative site for the measurement of glucose in diabetic and also in critically ill patients [11]. Several technologies have already been made commercially available for subcutaneous glucose monitoring [12, 13] and with lactate as an additional metabolic parameter, this minimally invasive technique could substantially enhance the field of application in clinical routine to improve prognosis and enable early therapeutic interventions. Patients recovering from major cardiac surgery are at high risk for complications and might benefit from a more close and accurate metabolic monitoring. Continuous subcutaneous lactate measurement could replace infrequent arterial measurement and consequently allow early therapeutic decisions to correct tissue oxygenation problems before they appear critical. However, careful evaluation of SAT versus blood lactate measurements using criteria as defined from a clinical perspective has not been performed to date. Therefore, the objective of the present study was first, to establish clinical evaluation criteria for SAT versus arterial blood lactate measurement, and second to investigate whether measurements from SAT could be used to replace arterial blood lactate measurements in patients admitted to an intensive care unit (ICU) after major cardiac surgery.

## 2. Materials and Methods

### 2.1. Patients

Forty adult patients from two different intensive care units were investigated after major cardiothoracic surgery (coronary artery bypass grafting 70%, valve replacement 15%, both 5%, aortic root replacement 10% of patients). Patients were included into the study after admission to the ICU for the duration of ICU stay but for a maximum period of 48 hours (mean: 42 hours, range: 15 to 48 hours). Exclusion criteria were (1) mental incapacity or language barriers precluding adequate understanding or cooperation and (2) any disease or condition which the investigator or treating physician felt would interfere with the trial or patient safety. Signed informed consent was obtained from all patients before surgery and before any trial-related activities. Patient characteristics are depicted in Table 1. The study was approved by the local Ethics Committee at Charles University Prague and at Medical University Graz.

### 2.2. Definition of Clinical Evaluation Criteria for Lactate Measurements

During clinical routine, measurement of absolute lactate levels, preferably from arterial blood, has been established as state-of-the-art technology. In addition, information about lactate trends (increase/decrease/stable) has been used to provide clinical information about the patient health status, clinical course, and prognosis factor. Therefore, for the present investigation, lactate concentrations were evaluated regarding levels of *absolute concentrations* and regarding *lactate trend information (change of lactate concentrations over time)*.

#### 2.2.1. Evaluation Criteria Based on Absolute Lactate Concentrations

**Figure 1:**
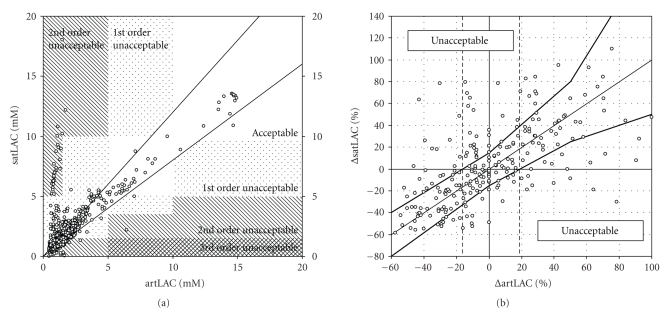
(a) Clinical evaluation of absolute lactate levels from subcutaneous adipose tissue (*sat*LAC) on the *y*-axis versus reference measurements from arterial blood (*art*LAC) on the *x*-axis. Clinical lactate grid as described in the method section is indicated as hatched areas. (b) 6-hour lactate trend analysis with respective clinically acceptable and unacceptable ranges as described in the method section. Relative 6-hour lactate trend of reference arterial blood lactate readings (Δ *art*LAC) and calibrated subcutaneous lactate readings (Δ *sat*LAC) are indicated on *x*-axis and *y*-axis, respectively. Upper and lower thick lines represent limits for acceptable lactate trend identification from subcutaneous adipose tissue measurements. Vertical-hatched lines indicate thresholds for decreasing and increasing reference lactate concentrations.

Clinical thresholds were defined based on data from literature showing that significant increments in mortality rates are observed when specific cut-off values for blood lactate are crossed [4–9]. The definition of selected thresholds and the zones according to the severity of violation were acknowledged by clinical experts from participating centers before data analysis. Subcutaneous adipose tissue lactate (*sat*LAC) measurements were defined as acceptable if localized in the same concentration range between two threshold limits of arterial reference lactate readings (*art*LAC). A violation was defined when *sat*LAC and *art*LAC were not in the same range, what would result in erroneous treatment guidance. Lactate concentrations below 1.5 mM were considered normal [8]. Blood lactate concentrations ranging between 1.5 and 5 mM were defined as pathologically elevated, with an additional threshold defined at 3.5 mM [6]. Lactate concentrations in this range are already associated with a significantly higher risk of mortality for several critically ill patient populations. Lactate concentrations ranging between 5 and 10 mM have been associated with a substantially higher risk of death in the ICU and were therefore defined as concentrations within a state of emergency with request for further diagnosis and therapeutic intervention. Finally, lactate concentrations above 10 mM have been associated with extremely high risk of death and therefore represent a life threatening condition for a patient at the ICU. After predefinition of these thresholds, a standard *xy*-graph with reference arterial blood lactate readings on the *x*-axis and SAT lactate readings on the *y*-axis including the predefined ranges on the respective axis was established (Figure 1(a)). A general error for SAT lactate readings of ±20% was accepted. Clinical experts were then asked to define zones indicating acceptable and 1st, 2nd, and 3rd order of unacceptable lactate measurement.

#### 2.2.2. Evaluation Criteria Based on Lactate Trend Analysis

The following criteria were defined to evaluate whether the lactate trend (increase/decrease or stable lactate concentration) as measured in arterial blood can be replaced by measurements from SAT. A 6-hour time interval was defined as clinically relevant for lactate trend evaluation. We arbitrarily defined a 6-hour interval as average-time period for clinical decision making and as adequate to detect significant changes in the clinical status of a patient. Stable lactate concentrations (over a period of 6 hours) were defined within ±20% relative change of the arterial blood lactate concentration as indicated by vertical-dashed lines in Figure 1(b). Clinical experts were then asked to define the maximum acceptable relative error for underestimation and overestimation of specific lactate changes, that is, a 60% decrease, no change, a 50% increase, and a 100% increase of the reference lactate concentration within 6 hours of time. The graph shown in Figure 1(b) indicates the relative change of the lactate concentration in arterial blood on the *x*-axis and in SAT on the *y*-axis and includes the range for acceptable lactate trends as measured in SAT (area between upper and lower thick lines).

### 2.3. Protocol

Characteristic information about the patients including demographic data, medical history, concomitant medication, body composition, vital signs, and laboratory analysis from routine laboratory assessment was obtained during a screening visit prior to surgery. After admission to the ICU, patients were included into the trial. For sampling of interstitial fluid from SAT standard microdialysis technique was applied. A microdialysis catheter (CMA 60, Stockholm, Sweden) was inserted on the left or right side of the periumbilical region. Abdominal location of the microdialysis catheter was chosen as the easiest accessible place, not disturbing any surgical or monitoring procedures. The catheter was connected to a microinfusion pump (CMA 107, Stockholm, Sweden) [15, 16] and was constantly perfused at a flow rate of 1 *μ*L/minute with an isotonic solution of 5% mannitol [17]. At least 60 minutes after insertion of the microdialysis catheter experiments were started and dialysate was continuously sampled and collected in hourly fractions (E.G. from TIME 0800 until 0900) in interstitial sampling vials (CMA, Stockholm, Sweden). Arterial blood samples were collected in hourly intervals (E.G. at TIME 0800 and 0900), at the same time when SAT sampling vials were changed. Experiments ended at the latest 48 hours after start of the study or when patients were transferred from the ICU upon decision of the treating physician.

### 2.4. Analysis

Arterial blood lactate concentrations were measured by routine blood gas analysis using standard point-of-care testing devices (Graz: Omni S, Roche Diagnostics, Basel, Switzerland; Prague: ABL 700, Radiometer Medical, Copenhagen, Denmark). Samples from SAT dialysate were stored at −70°C immediately after sampling and were analyzed at a central laboratory (Joanneum Research GesmbH, Graz, Austria). Dialysate samples from subcutaneous adipose tissue were analyzed for lactate using a Cobas Mira Analyzer (Roche Diagnostics, Basel, Switzerland) using standard enzymatic assays (Roche Diagnostics GmbH, Mannheim), and sodium and potassium using a flame photometer (Instrumentation Laboratory GmbH, Vienna, Austria) after the finalization of the study at both participating centers. Intrarun (interrun) coefficients of variation were 5.8% to 1.0% (10% to 0.9%) and 1.7% to 0.4% (1.5% to 0.4%) for lactate and sodium for limit of quantification to maximum measurement range, respectively.

### 2.5. Calculations and Calibration

Data analysis of SAT lactate concentrations was based on fractionized samples of dialysate lactate concentrations from subcutaneous adipose tissue (*dia*LAC). Corresponding hourly measurements of arterial blood lactate (*art*LAC) were averaged between beginning (E.G. TIME 0800) versus end (E.G. TIME 0900) of the interstitial sampling interval. Lactate in SAT dialysate (*dia*LAC) was calibrated using a prospective 2-step calibration approach, first using the ionic reference technique, based on the correction of *dia*LAC, by multiplying with the ratio of sodium in interstitial fluid by blood levels, as previously indicated in greater detail [17, 18], to calculate the actual lactate concentration in SAT and second, using a one-point calibration procedure to obtain a lactate profile from SAT comparable to arterial blood lactate concentrations (*dia*LAC $\overset{2\text{-step calibration}}{\to }$ *sat*LAC). As a second calibration step, a one-point calibration procedure was applied. Using this procedure, the ratio between the first lactate reading from SAT and the average of the two corresponding blood lactate readings (*art*LAC) was used to calculate the SAT derived lactate profile (*sat*LAC). One-point instead of multiple point calibration was chosen to establish a critical evaluation of SAT derived lactate measurements [19].

### 2.6. Statistical Analysis

Normal distribution of data was tested using Shapiro-Francia test. Bland and Altman analysis was used to compare arterial with prospectively calibrated interstitial lactate concentrations. *P*-values smaller than 0.05 were considered statistically significant.

## 3. Results

Application of the microdialysis procedure in subcutaneous adipose tissue was well tolerated and no adverse events related to the interstitial sampling procedure occurred during or after the experiments. Forty critically ill patients were investigated for a period of 41.9 ± 12.1 hours (mean ±SD) and a total of 1550 paired lactate measurements were analyzed to clinically evaluate the relation between arterial blood and subcutaneous adipose tissue (SAT) lactate measurements. Noradrenalin was used during the ICU stay in 25 patients (63%), whereas dobutamin was used in 7 patients (18%) at doses of 0.09 ± 0.08 *μ*g/kg/minute and 4.9 ± 2.4 *μ*g/kg/minute, respectively.

### 3.1. Clinical Evaluation Based on Absolute Lactate Concentrations

For the evaluation of absolute interstitial lactate measurements 1550 paired readings were considered. The distribution of arterial blood lactate readings was as follows, 57.7% were found in the normal range (<1.5 mM), 38.1% were found in the range between 1.5 and 5 mM, 2.8% of the readings were found in the critical range >5 mM, and <10 mM and 1.4% of the readings were found above 10 mM. Clinical evaluation of arterial blood (*art*LAC) versus calibrated SAT lactate (*sat*LAC) readings is depicted in Figure 1(a). According to the approximate precision of arterial lactate measurement using laboratory standards, less than 5% of the measurements in the unacceptable zone have been considered as reliability criteria for the method. A total of 76.5% or 1186 readings were found in the acceptable measurement zone, 17.6% or 273 readings in the first-order unacceptable zone, and 5.9% or 91 readings from SAT in the second-order unacceptable zone (Table 2). It can be summarized that for 23.5% or 364 lactate readings from SAT, no reliable information about the actual reference arterial blood lactate concentration can be obtained.

To test whether a 6-hour stabilization period of the microdialysis system would improve the relation between SAT and arterial lactate readings we performed a different calibration approach, that is, we directly calibrated SAT lactate to the arterial blood concentration at hour 6 instead of hour 1 and considered only the data from hour 6 to 48 for clinical evaluation. This modified procedure, improved the relation between SAT and arterial blood, yet the results indicate that still 5.5% of the readings were in the unacceptable measurement zone (see Table 4).

### 3.2. Clinical Evaluation Based on 6-Hour Lactate Trends

For the evaluation of 6-hour lactate trends, a total of 245 paired trends were provided by the analysis and compared between reference trends in arterial blood (Δ *art*LAC) and trends from SAT (Δ *sat*LAC). The overall distribution of lactate trends with the respective clinically acceptable and unacceptable ranges is indicated in Figure 1(b). Most of the overall lactate trends (65.3%) as measured in SAT were in the acceptable measurement zone; however, 34.7% were in the unacceptable zone. Results describing the capability of SAT lactate to identify stable, increasing and decreasing arterial lactate trends are summarized in Table 3. In summary, the 6-hour lactate trend analyses indicated that about a third of the 6-hour lactate trends from arterial blood would be falsely identified by SAT lactate measurements and would provide an unacceptable violation of the clinical interpretation of the trend information. The analysis performed with calibration after a 6-hour stabilization period for the microdialysis catheter indicated that 41.6% of the measurements were in the unacceptable zone, as summarized in Table 5.

### 3.3. Examples for Comparison of Lactate Time Profiles

**Figure 2:**
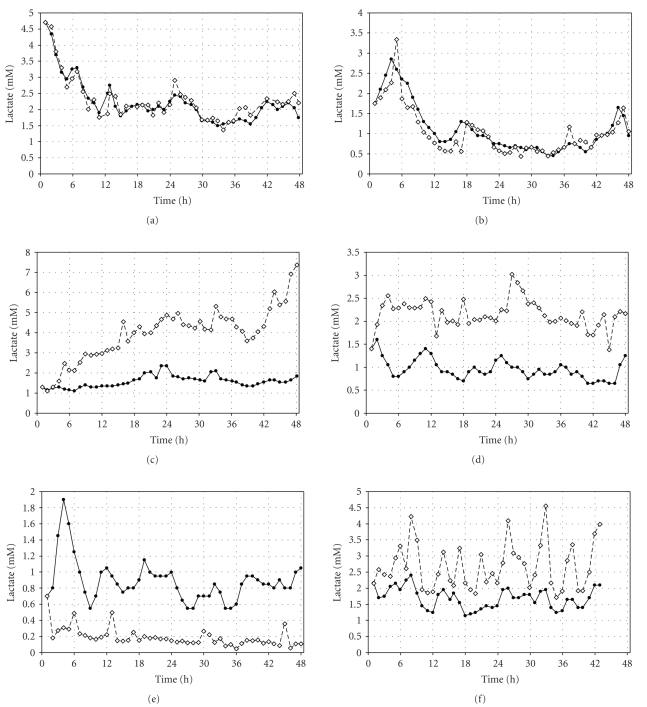
Time profiles for individual patients. Arterial lactate (closed symbols) versus subcutaneous adipose tissue lactate (open symbols) concentrations. Panels indicate examples with different levels of interrelation between arterial and subcutaneous adipose tissue lactate readings.

Figure 2 indicates time profiles for individual patient comparisons for *art*LAC versus *sat*LAC readings using a prospective one-point calibration procedure. Upper panels indicate examples with best relation, middle and lower panels' results with worst relation between *art*LAC and *sat*LAC readings according to clinical evaluation of absolute lactate readings and 6-hour lactate trend analysis, respectively.

## 4. Discussion

Clinical evaluation of lactate measurements from SAT in critically ill patients after major cardiosurgical operation using the method of microdialysis indicated that replacement of arterial blood lactate measurements by readings from SAT is associated with a substantial shortcoming in clinical predictability in patients after major cardiac surgery.

Hyperlactatemia has clearly been associated with poor clinical outcome [4–9] and therefore, the definition of different lactate ranges for clinical evaluation of lactate measurements from SAT was based on studies investigating the relation between arterial blood lactate concentrations and clinical outcome parameters. An important threshold for the arterial blood lactate concentration in the critically ill patient is the threshold between lactate normal (below 1.5 mM) and lactate high (above 1.5 mM). The majority of the lactate readings of the present study were found around this threshold level. Whereas the predictability of blood lactate using SAT measurements was relatively good for very high lactate concentrations above 5 mM, it was only poor around the threshold of 1.5 mM, where a substantial amount of SAT lactate readings over- and underestimated the reference blood lactate concentration suggesting that especially within this critical range, SAT lactate measurements failed to provide a replacement for arterial blood lactate readings.

From a clinical monitoring point of view, for example, to guide a therapy, it is important to follow the actual measured lactate concentration in form of a lactate profile (trend) over time [20]. Increasing lactate concentrations over time may indicate a deteriorating health condition and the requirement of additional diagnostic action or therapy or may indicate the failure of a given therapeutic action, whereas a decrease of high lactate concentrations towards lactate normal states below 1.5 mM may be considered as an improvement in health condition or that the applied therapeutic action has been successful. For the evaluation of lactate trend information, 48-hour arterial lactate profiles from the present investigation have been evaluated. Intensive care physicians defined a 6-hour measurement interval as clinically relevant for the repeated measurement of lactate concentrations within a single patient. Applying the data of the present study to the clinical trend analysis indicated that approximately one third of the 6-hour lactate trends from SAT would not allow an accurate prediction of the given change of the arterial lactate concentration. More than 25% of the analyzed lactate increases (>20%) from SAT would be falsely identified as stable or even decreasing lactate concentrations. This result clearly demonstrates, that as applied in the present experiments, lactate readings from SAT cannot accurately predict the trend information of arterial blood lactate readings in critically ill patients after major cardiovascular surgery.

Reasons for these observations might be of physiological or methodological origin. Individual lactate profiles as depicted in Figure 2 suggest that both explanations could have contributed to our findings. The divergent behavior of SAT lactate in comparison to arterial blood lactate to a continuous increase as shown in the left middle panel in Figure 2 can possibly be explained by lack of tissue perfusion, causing tissue hypoxia. This could lead to enhanced anaerobic conditions and pronounced lactate production in SAT, causing only a mild systemic increase of the lactate concentration. In contrast, the initial rise in SAT lactate as seen in the right middle panel of Figure 2 could most likely be explained by an initial microdialysis instability after insertion of the catheter as frequently seen in previous observations using this technique [11, 21, 22]. Another important argument in favor of physiological reasons for the present observation is the fact that in the same patient population and using the same technology, *glucose* readings from arterial blood could very well be described by readings from SAT [11]. In contrast, for SAT lactate, only for ~50% of the patients, a reliable relation between SAT and arterial blood was found and no other parameter such as severity of illness (APACHE II), BMI, age, systemic blood pressure, heart rate, medication, or the interstitial glucose concentration were able to explain the difference between these patients. Methodological reasons could be considered to explain the diversity of lactate profiles, as for example, a local infection at the catheter insertion site, leading to increased local oxygen consumption, or instability of the microdialysis exchange properties, initially after catheter insertion. A distinction between these two effects is extremely difficult and would require a substantially higher number of patients studied.

## 5. Limitations and Possible Improvements

Limitations of the methodological approach of microdialysis as used in the present study could contribute to the lack of association between arterial blood and SAT lactate readings. Different sites of subcutaneous monitoring might provide different reproducibility profiles in comparison to the observed results. Local tissue perfusion which is known to be associated with the severity of illness of an individual patient may also be associated with local adipose tissue lactate release. Although no such correlation was observed in the present investigation, this may also be due to the relatively small group of patients included. Also, it seems that different calibration techniques (calibration immediately after catheter insertion versus 6-hour calibration after catheter insertion) affect clinical reliability of subcutaneous lactate readings. In light of these observations, further studies, with a special focus on patient populations with a wide range in severity of illness with regard to circulatory failure and instability will allow further insight into this rather complex topic.

## 6. Conclusion

Minimally invasive techniques to access subcutaneous adipose tissue for glucose monitoring have already been successfully applied in type 1 diabetic and in critically ill patients. Especially in the critically ill patient setting, the addition of a lactate sensor, that is, the combined measurement of glucose and lactate might substantially enhance the clinical application field of such a technology. However, the results of the present study clearly indicate that using the applied technology and calibration approach, lactate readings from SAT are associated with a substantial shortcoming in clinical predictability in patients after major cardiac surgery.

## Figures and Tables

**Table 1:** Baseline characteristics.

	Graz	Prague	Total
Patients [*n*]	20	20	40
Age [years]	68.6 ± 7	66.0 ± 11	67.3 ± 9
Female [*n*]	5	3	8
Ethnicity: Caucasian [*n*]	20	20	40
BMI [kg/m^2^]	28.2 ± 4.9	27.0 ± 4.0	27.6 ± 4.4
History of Diabetes [*n*]	6	10	16
BP syst. [mmHg]	108 ± 10	119 ± 10	114 ± 11
BP diast. [mmHg]	54 ± 5	57 ± 5	55 ± 5
Heart Rate	90 ± 10	88 ± 7	89 ± 9
APACHE II^§^ score	10.1 ± 3.2	11.4 ± 4.5	10.7 ± 3.9

Data are mean ±SD, [*n*] number of patients; ^§^Acute physiology and chronic health evaluation II score [14].

**Table 2:** Clinical evaluation of absolute subcutaneous adipose tissue versus arterial blood (*art*LAC) lactate concentrations stratified for individual lactate ranges according to Figure 1(a).

*n* = 1550 hourly paired lactate readings	Acceptable %	Unacceptable [1st order] %	Unacceptable [2nd order] %
*art*LAC < 1.5 mM	77.6	22.2	0.2
1.5 mM ≤ *art*LAC < 3.5 mM	72.9	12.2	14.9
3.5 mM ≤ *art*LAC < 5 mM	80.0	20.0	0.0
5 mM ≤ *art*LAC < 10 mM	95.4	2.3	2.3
10 mM ≤ *art*LAC	95.2	4.8	0.0

Total readings	76.5	17.6	5.9

No readings from subcutaneous adipose tissue were found within the third-order unacceptable measurement zone.

**Table 3:** Clinical evaluation of 6-hour lactate trend analysis from subcutaneous adipose tissue (Δ *sat*LAC) versus arterial blood (Δ *art*LAC). Lactate measurements are stratified to positive, stable and negative lactate trends as indicated in Figure 1(b).

*n* = 245 6-hourly paired lactate trends	Acceptable measurement %	Unacceptable falsely high %	Unacceptable falsely low %
Positive trend (>20%)	61.9	11.1	27.0
Negative trend (< −20%)	66.7	27.0	6.3
No change (±20%)	66.4	20.9	12.7

Total trend analysis	65.3	19.9	14.8

**Table 4:** Clinical evaluation of absolute subcutaneous adipose tissue versus arterial blood (*art*LAC) lactate concentrations stratified for individual lactate ranges after 6-hour one-point calibration.

*n* = 1369 hourly paired lactate readings	Acceptable %	Unacceptable [1st order] %	Unacceptable [2nd order] %
*art*LAC < 1.5 mM	96.1	3.9	0.0
1.5 mM ≤ *art*LAC < 3.5 mM	90.9	9.1	0.0
3.5 mM ≤ *art*LAC < 5 mM	100.0	0.0	0.0
5 mM ≤ *art*LAC < 10 mM	96.6	3.4	0.0
10 mM ≤ *art*LAC	100.0	0.0	0.0

Total readings	94.5	5.5	0.0

**Table 5:** Clinical evaluation of 6-hour lactate trend analysis from subcutaneous adipose tissue (Δ *sat*LAC) versus arterial blood (Δ *art*LAC) after 6-hour 1-point calibration. Lactate measurements are stratified to positive, stable and negative lactate trends.

*n* = 231 6-hour paired lactate trends	Acceptable measurement %	Unacceptable falsely high %	Unacceptable falsely low %
Positive trend (>20%)	60.0	12.3	27.7
Negative trend (< −20%)	59.2	29.6	11.3
No change (±20%)	56.7	24.7	18.6

Total trend analysis	58.4	22.7	18.9
